# Metformin suppresses UHMWPE particle-induced osteolysis in the mouse calvaria by promoting polarization of macrophages to an anti-inflammatory phenotype

**DOI:** 10.1186/s10020-018-0013-x

**Published:** 2018-05-09

**Authors:** Zhao Yan, Xiaoxi Tian, Jinyu Zhu, Zifan Lu, Lifeng Yu, Dawei Zhang, Yanwu Liu, Chongfei Yang, Qingsheng Zhu, Xiaorui Cao

**Affiliations:** 1 PLA Institute of Orthopaedics, Xijing Hospital, Fourth Military Medical University, Xi’an, 710032 China; 2 Emergency department of Tangdu Hospital, Fourth Military Medical University, Xi’an, 710038 China; 3 State Key Laboratory of Cancer Biology, Department of Pharmacogenomics, Fourth Military Medical University, Xi’an, 710032 China

**Keywords:** Inflammation, Osteoclasts, AMPK, Macrophage, Osteolysis

## Abstract

**Background:**

Implant failure remains a major obstacle to successful treatment via TJA. Periprosthetic osteolysis and aseptic loosening are considered as proof of wear debris-induced disruption of local regulatory mechanisms related to excessive bone resorption associated with osteolysis and the damage at the bone-prosthesis interface. Therefore, there is an immediate need to explore strategies for limiting and curing periprosthetic osteolysis and aseptic loosening.

**Methods:**

We analyzed the in vitro cytokine production by primary mouse bone marrow macrophages (BMMs) that were exposed to ultra-high molecular weight polyethylene (UHMWPE) particles and treated with metformin at different concentrations with or without 5-aminoimidazole-4-carboxamide ribonucleoside to activate or inhibit AMPK. A mouse calvarial model was used to examine the in vivo effects of metformin on UHMWPE particle-induced osteolysis.

**Results:**

With particles, primary mouse BMMs secreted more pro-inflammatory cytokines tumor necrosis factor-α and interleukin (IL)-6. Treatment with metformin inhibited these variations and promoted the release of cytokine IL-10 with anti-inflammatory capability. In vivo, metformin reduced the production of pro-inflammatory cytokines, osteoclastogenesis, and osteolysis, increasing IL-10 production. Metformin also promoted the polarization of macrophages to an anti-inflammatory phenotype in vivo via AMPK activation.

**Discussion:**

A crucial point in limiting and correcting the periprosthetic osteolysis and aseptic loosening is the inhibition of inflammatory factor production and osteoclast activation induced by activated macrophages. The ability of metformin to attenuate osteolysis induced in mouse calvaria by the particles was related to a reduction in osteoclast number and polarization of macrophages to an anti-inflammatory functional phenotype.

**Conclusions:**

Metformin could limit the osteolysis induced by implant debris. Therefore, we hypothesized that metformin could be a potential drug for osteolysis induced by implant debris.

**Electronic supplementary material:**

The online version of this article (10.1186/s10020-018-0013-x) contains supplementary material, which is available to authorized users.

## Background

Total joint arthroplasty (TJA) is a widely performed surgery for reducing the pain and restoring the mobility of patients with major joint damage or severe arthritis. Implant failure remains a major obstacle to successful treatment via TJA. Osteolysis induced by implant wear debris is the dominant cause of implant failure, causing ~ 50,000 revision surgeries per year in the United States alone (Kurtz et al. 2007). Long-range data regarding TJA outcomes are insufficient, and osteolysis continues to be a common complication. Therefore, there is an immediate need to explore strategies for limiting and curing periprosthetic osteolysis and aseptic loosening.

Osteolysis begins with the activation of macrophages in addition to foreign-body giant cells and the phagocytosis of particulate wear debris. These events stimulate the release of cytokines with pro-inflammatory capability and mediators and reduce the phosphorylation of AMP-activated protein kinase (AMPK) (Vasamsetti et al. 2015), promoting the production of osteoclasts that are differentiated from phagocyte precursors and in turn facilitating the periprosthetic osteolysis and corresponding implant loosening (Glant et al. 1993). Osteoblast activity is hampered by exposure to prosthetic particles. For example, osteoblasts expressed a catabolic phenotype upon exposure to polyethylene particles (Atkins et al. 2009). Periprosthetic osteolysis and aseptic loosening, thus, are considered as proof of wear debris-induced disruption of local regulatory mechanisms related to excessive bone resorption associated with osteolysis and the damage at the bone-prosthesis interface (Purdue et al. 2006). A crucial point in limiting and correcting the periprosthetic osteolysis and aseptic loosening, thus, is the inhibition of inflammatory factor production and osteoclast activation induced by activated macrophages.

As an anti-hyperglycemic agent, metformin is widely used in treating type II diabetes to improve insulin resistance (Inzucchi et al. 2014). Metformin has a bone-protective property (La Fontaine et al. 2016) and enhances the mineralization and differentiation of osteoblasts (Kanazawa et al. 2008), negatively regulating RANKL in osteoclast differentiation (Lee et al. 2010). The reason is related to the activation of a serine/threonine protein kinase of AMP-activated protein kinase (AMPK), which is critical in maintaining metabolic homeostasis in eukaryotic cell types (Kudo et al. 1995). The anti-inflammatory effect of metformin is associated with AMPK phosphorylation. In addition, AMPK promoted the polarization of macrophages to an anti-inflammatory functional phenotype (Sag et al. 2008). Therefore, we hypothesized that metformin could limit the osteolysis induced by implant debris. Here, how metformin affects the in vitro production of inflammatory factors in primary mouse bone marrow macrophages (BMMs) in response to ultra-high molecular weight polyurethane (UHMWPE) particles was investigated. The ability of metformin to attenuate osteolysis induced in mouse calvaria by the particles was examined and found to be related to a reduction in osteoclast number and polarization of macrophages to an anti-inflammatory functional phenotype.

## Methods

### Preparation of UHMWPE-coated coverslips

The average diameter of the UHMWPE particles (provided by Mr. Ernst Krendlinger of Clariant, Gersthofen, Germany) was 1.74 ± 1.43 μm (range, 0.05–11.06 μm), with 90% particles smaller than 9 μm. (The particle diameter in membranes around total joint prostheses ranged from approximately 0 to 1 μm.) Notably, this particle diameter is within the range considered biologically active and differs from the diameter of particles isolated from the periprosthetic membranes. All particles were rinsed to remove the endotoxins using ethanol before desiccation in drying oven, and correspondingly, tests for endotoxins using the Limulus assay (Sigma-Aldrich, St. Louis, MO, USA) were negative. Before use in cell culture, the particles were disinfected under γ-irradiation in air (2.5 MR) and kept at 4 °C. The particles were exposed to UV light for 1 h immediately before use.

Glass coverslips were coated with UHMWPE particles. Previously characterized UHMWPE particles were placed in dimethyl sulfoxide (DMSO; 1 mg particles in 0.5 mL DMSO) in a sterile quartz pestle and mortar. Subsequently, the materials were suspended in 14.5 mL of collagen type-I monomer (0.01%) solution prepared from calf skin (C-8919; Sigma-Aldrich) and maintained at 4 °C. The final particle concentration was set at 10^7^ particle/mL. The microscope coverslips with dimensions of 22 × 22 mm (Dingjie Biological Technology Co., Ltd., Shanghai, China) were disinfected for 2 h at 200 °C. Aliquots of collagen-suspended particles (10^5^ particles/10 μL) were distributed onto the coverslip uniformly, before collagen polymerization was allowed to occur at room temperature. Coverslips not coated with UHMWPE were also created and used in control samples. The coverslips were placed into 6-well tissue culture plates before irradiation with ultraviolet light overnight in a sterile tissue culture hood. The plates were placed in PE packages and covered with aluminum foil. The storage temperature was 5 °C. Another 1 h exposure was performed immediately before usage of the coverslips (Nich et al. 2011).

### Cell culture and IL-6, and TNF-α measurements

Dulbecco’s Modified Eagle Medium (DMEM) supplemented with 10% fetal bovine serum (FBS; Gibco) and penicillin (100 U/mL) was used for primary mouse BMM culture, and the cells were obtained from mouse femurs as described previously (Shah et al. 2010). RPMI-1640 supplemented with streptomycin (100 mg/mL), 10% FBS (Gibco), penicillin (100 U/mL), 10% heat-inactivated fetal calf serum, and L-glutamine (2 mM) was used for RAW264.7 cell culture, and the cells were obtained from American Type Culture Collection (Rockville, MD, USA).

The cells were seeded at 1 × 10^6^ cells/2 mL/well and exposed to UHMWPE particles on the coated coverslips for 24 h in 6-well tissue culture plates. To examine how the particles affected both cells, the cells were treated with metformin at a concentration of 0.05, 0.5, or 5 mM with or without various concentrations of compound C (Sigma-Aldrich), a chemical inhibitor of AMPK, as well as 5-aminoimidazole-4-carboxamide1-β-D-ribofuranoside (AICAR, Sigma-Aldrich), which stimulates AMPK activity. The control samples were exposed to deionized water and incubated for 24 h. After each incubation period, the media samples were collected for cytokine measurement. Enzyme-linked immunosorbent assay (ELISA) kits from R&D Systems (Minneapolis, MN, USA) were used to measure the concentrations of tumor necrosis factor (TNF)-α, IL-6, and IL-10. Cells were lysed and centrifuged at 1000 g at 4 °C for 5 min and then at 10,000 g at 4 °C for 10 min. After the supernatants were collected, ARG-1 in the supernatants was quantified using an ELISA kit (LifeSpan BioSciences) according to the manufacturer’s instructions.

### Animals and surgical procedure

Ninety 12-week-old C57BL/J6 male mice were utilized to establish the calvarial model of particle-induced osteolysis. All experiments involving animals were approved by the Institutional Review Board of Xijing Hospital (permit No. 20161003). All methods were performed in accordance with National Institute of Health Guide for the Care and Use of Laboratory Animals. The mice were divided into four groups randomly (*n* = 22/group). Five mice in each group were used to collect calvaria for tissue culture, five for RNA extraction and qRT-PCR, five for Western blot analysis, and the other seven for histologic evaluation after micro-computed tomography scanning. For surgery, mice were placed under general anesthesia via inhalation of isoflurane. An area (0.5 × 0.5 cm^2^) of the periosteum was exposed via a midline sagittal incision of 10 mm on the calvaria. The incision in the sham control was sutured without further procedure. In the experimental groups in which UHMWPE particles were implanted, a sterile sharp surgical spoon was used to uniformly distribute 20 μg of particles over the intact periosteum. A simple, interrupted 4-0 Ethicon suture was utilized to close the incision. The mice were subsequently placed into cages and provided food and water ad libitum. All animals were given metformin (80 mg/kg, once a day for 2 weeks in a row), alendronate (ALN, Sigma-Aldrich, as positive control, 50 μg/kg, once a day for 2 weeks as previously described as anti-osteoporosis use), or normal saline as negative control via intragastric administration immediately after surgery. The animals were sacrificed 14 days after the operation via an overdose of intraperitoneal sodium pentobarbital.

### Characterizations

The detailed descriptions of micro-CT analyses, histologic evaluation of osteolysis, quantitative real-time polymerase chain reaction (qRT-PCR), calvaria culture, and western blot analysis are provided in Additional file 1: Supporting Information S3.

### Statistical analysis

All experiments were performed at least three times. All data are presented as mean ± standard deviation (SD). The SPSS software package (SPSS 11.0, Chicago, IL, USA) was used for statistical analyses. One-way analysis of variance (ANOVA) was performed to compare the different groups. Post-hoc testing of discrepancies among groups was performed via Duncan’s test when the ANOVA results were significant. All *P* values < 0.05 were considered indicative of significance.

## Results

### Effects of metformin on TNF-α, IL-6 and IL-10 production and macrophage transition in primary mouse BMMs

**Fig. 1:**
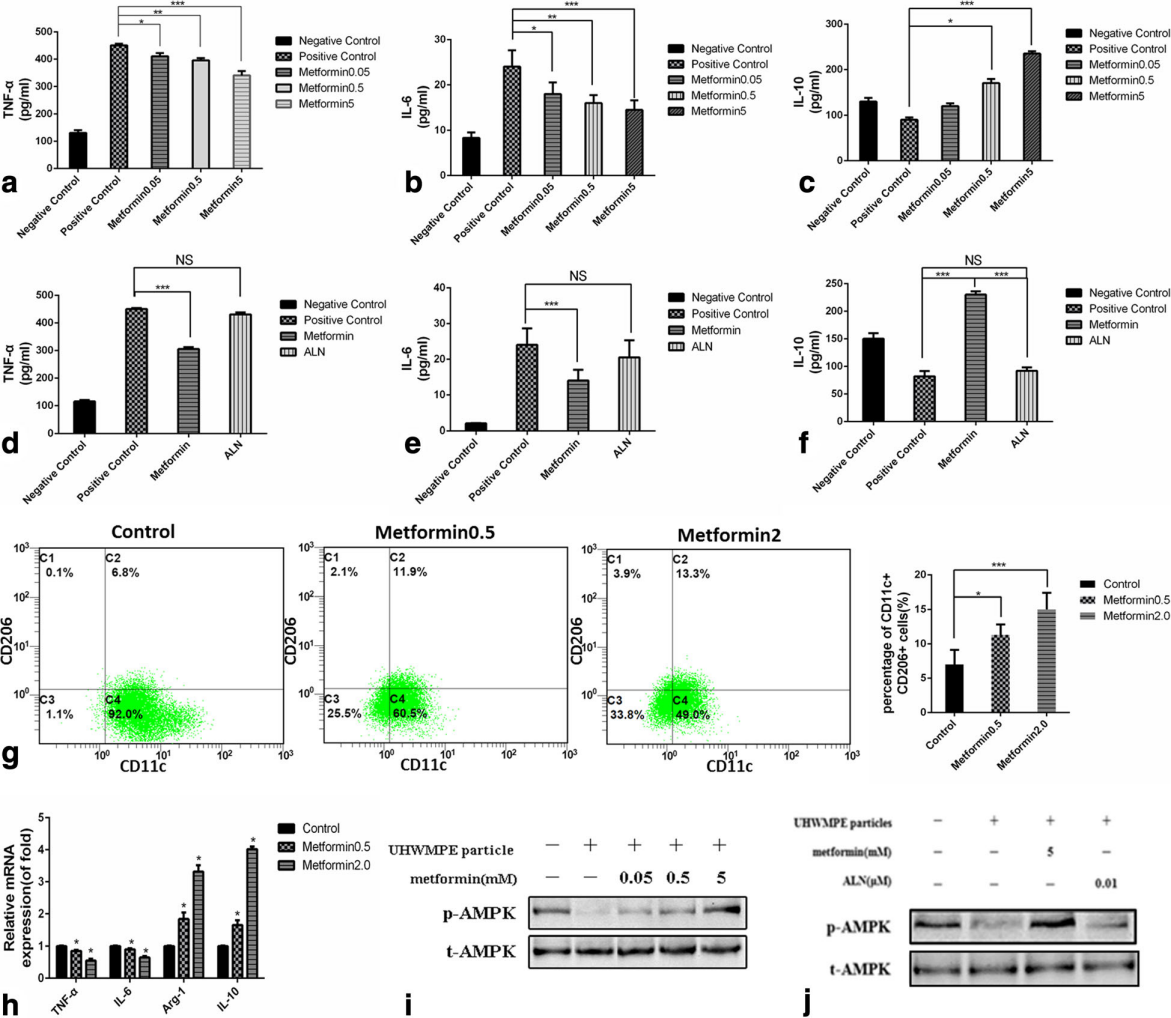
Effect of metformin on primary mouse BMM cytokine production in response to UHMWPE particles and the effect on AMPK phosphorylation. Concentrations of (**a**) TNF-α, (**b**) IL-6, and (**c**) IL-10 in culture media after exposure of the cells to UHMWPE particles and various concentrations (0.05, 0.5, or 5 mM) of metformin. Concentrations of (**d**) TNF-α, (**e**) IL-6, and (**f**) IL-10 in culture media after exposure of the cells to UHMWPE particles and 5 mM metformin as well as 0.01 μM ALN. **g** Flow cytometric analysis of control and metformin-treated groups. **h** QRT-PCR results for M1 marker mRNA expression and M2 marker mRNA expression. **i**–**j** Western blot results for phosphorylated AMPK (p-AMPK) and total AMPK (t-AMPK) in the different treatment groups. (Cropped gels/blots are used here, and the full-length gels and blots are shown in Additional file 1: Figure S3.) Data represent the means ± SD. NC, negative control (no treatment); PC, positive control (treatment with UHMWPE particles only). ***P < 0.001; **P < 0.01; *P < 0.05

Production of IL-6 and TNF-α as pro-inflammatory mediators, as well as IL-10 as an anti-inflammatory mediator by primary mouse BMMs exposed to UHMWPE particles for 24 h was measured using ELISA kits. Within the incubation period of 24 h, the TNF-α and IL-6 levels in the media increased significantly (*P* < 0.05), greater than those in the control cultures not exposed to particles (Fig. 1a–b). In contrast, the IL-10 levels in the culture media did not differ obviously between the groups (Fig. 1c). Treatment with metformin significantly reduced the increase in production of both TNF-α and IL-6, but enhanced the release of IL-10 after exposure to the particles for 24 h in a dose-dependent manner (Fig. 1a–c). ALN is widely used for the prevention and treatment of osteoporosis in postmenopausal women and has been shown to increase bone mass in men with osteoporosis (Kostenuik et al. 2015). ALN is used here as a positive control to facilitate the understanding of the mechanisms of metformin’s action. ALN cannot inhibit the production of TNF-α and IL-6, nor can it enhance the release of IL-10 (Fig. 1d–f; *P* < 0.05). We also investigated RAW264.7 cells and obtained similar results (see Additional file 1: Supporting Information S1 and Figure S1). Moreover, flow cytometric results showed polarization of macrophages to the M2 phenotype upon treatment with UHMWPE particles and metformin (Fig. 1g). QRT-PCR results also showed reduced M1 marker mRNA expression and increased M2 marker mRNA expression (Fig. 1h).

### Role of AMPK activation in the effect of metformin on the exposed primary mouse BMMs

Metformin inhibition of inflammatory cytokine by lipopolysaccharide (LPS)-treated macrophages depended on the AMPK phosphorylation (Musi et al. 2002; Tsoyi et al. 2011). Whether AMPK activation played a role in the effect of metformin on cytokine release by primary mouse BMMs exposed to UHMWPE particles was examined. Immunoblot analysis demonstrated negative regulation of phosphorylated AMPK with particle exposure, whereas the expression of total AMPK was not significantly altered upon exposure to the particles (Fig. 1i). The inhibition of AMPK phosphorylation in primary mouse BMMs with particles was recovered by metformin also in a dose-dependent manner. This effect was similar to that observed in relation to cytokine release. However, AMPK phosphorylation was not affected by ALN (Fig. 1j). Metformin thus stimulated the activation of AMPK in macrophages exposed to UHMWPE particles.

**Fig. 2:**
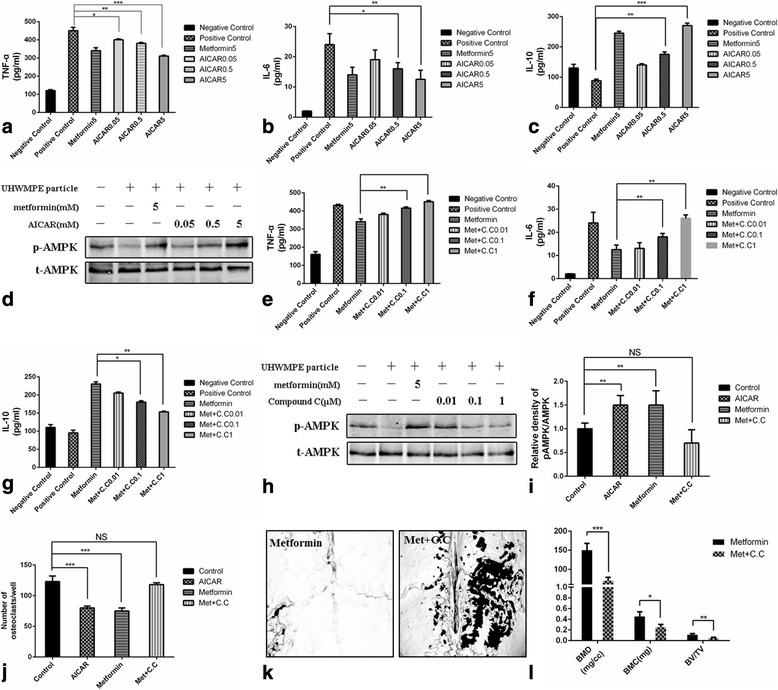
Concentrations of (**a**) TNF-α, (**b**) IL-6, and (**c**) IL-10 in culture media after exposure to UHMWPE particles, metformin (5 mM), and different concentrations of AICAR (0.05, 0.5, or 5 mM) to activate AMPK. **d** Western blot results for phosphorylated AMPK (p-AMPK) and total AMPK (t-AMPK) in the different treatment groups. (Cropped gels/blots are used here, and the full-length gels and blots are shown in Additional file 1: Figure S4.) Concentrations of (**e**) TNF-α, (**f**) IL-6, and (**g**) IL-10 in culture media after exposure to UHMWPE particles, metformin (5 mM), and different concentrations of Compound C (0.01, 0.1, or 1 μM) to block AMPK. **h** Western blot results for phosphorylated AMPK (p-AMPK) and total AMPK (t-AMPK) in the different treatment groups. (Cropped gels/blots are used here, and the full-length gels and blots are shown in Additional file 1: Figure S4.) **i** Relative ratio of pAMPK/AMPK. **j** Number of osteoclasts per well in different groups. **k** Micro-CT scans of mouse calvaria in metformin-treated group and metformin plus compound C group. **l** BMD, BMC, BV/TV of both groups. Data represent the means ± SD. NC, negative control (no treatment); PC, positive control (treatment with UHMWPE particles only), C.C, Compound C. ***P < 0.001; **P < 0.01; *P < 0.05

To investigate the dependence of cytokine production on AMPK activation by metformin, primary mouse BMMs were treated with AICAR to induce AMPK activation. In the same culture conditions, AICAR inhibited the particle-induced increases in TNF-α and IL-6 production and promoted IL-10 release in a concentration-dependent manner (Fig. 2a–c). Furthermore, as expected, AICAR treatment was associated with increased AMPK phosphorylation in a dose-dependent manner. The changes in AMPK phosphorylation with AICAR treatment mirrored those observed with metformin treatment (Fig. 2d). AMPK was blocked next using compound C as a chemical inhibitor (Li et al. 2015). Compound C not only limited the AMPK phosphorylation in a concentration-dependent manner, but clearly neutralized the effect of metformin on cytokine production (Fig. 2e–h). The effect of metformin on cytokine release, thus, was highly related to AMPK activation in primary mouse BMMs exposed to the particles. We next examined the effects of metformin on osteoclastic differentiation. Primary mouse BMMs were treated with PBS, metformin, AICAR, or metformin plus compound C and then stimulated with soluble recombinant RANKL (100 ng/ml) and M-CSF (30 ng/ml). After 7 days, we observed elevated pAMPK activity in the metformin- and AICAR-treated groups (Fig. 2i). Similarly, we observed reduced OC numbers in these two groups (Fig. 2j). Therefore, we inferred that metformin inhibited the differentiation of BMMs into osteoclasts by activating AMPK phosphorylation.

We also investigated in vitro cytokine production by RAW246.7 cells exposed to UHMWPE particles and treated with metformin at different concentrations with or without 5-aminoimidazole-4-carboxamide ribonucleoside to activate or inhibit AMPK (see Additional file 1: Supporting Information S2 and Figure S2). Similar results were obtained, which confirmed the influence of metformin.

### Metformin attenuated particle-induced mouse calvarial osteolysis without changing the blood glucose level

**Fig. 3:**
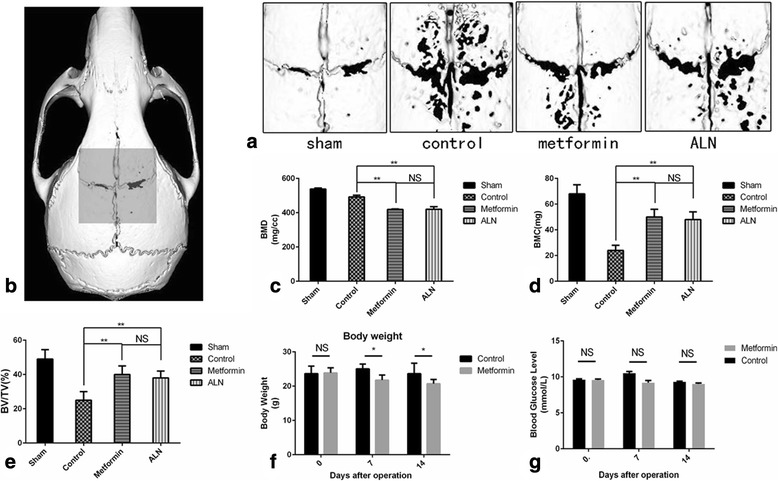
Metformin attenuated particle-induced mouse calvarial osteolysis without changing the blood glucose level. **a** Three-dimensional images from micro-CT scans of mouse calvaria, showing evidence of resorption pits along the suture lines in mice in which UHMWPE particles were implanted in comparison with representative images from the metformin- and ALN-treated groups. **b** ROI selected for quantitative analysis of BMD (**c**), BMC (**d**), and BV/TV (**e**) in each group. **f** Changes in body weight after operation at days 0, 7 and 14. **g** Comparison of blood glucose levels between groups ***P < 0.001; **P < 0.01; *P < 0.05; NS, not significant, *P* > 0.05

Mouse calvaria harvested 14 days after surgical implantation of UHMWPE particles were qualitatively and quantitatively analyzed by micro-CT (Fig. 3). The low signal area represents the sagittal suture area with neighboring bone resorption. Four groups were used to investigate the metformin effect. The area of bone resorption observed following implantation of the UHMWPE particles was reduced by treatment with metformin and ALN compared with that in mice that received no further treatment (Fig. 3a). From 3D reconstruction imaging, bone quantity and quality were evaluated according to the BMD, BMC, and BV/TV of ROIs (Fig. 3b). All three parameters were the lowest in the mice calvaria implanted with UHMWPE particles and given no further treatment, and the bone resorption reaction was evident. With daily metformin or ALN treatment, the BMD, BMC, and BV/TV of the calvaria were higher than those without additional treatment following particle implantation, whereas no significant differences were observed between the groups treated with metformin and ALN (Fig. 3c–e). At the same time, the metformin-treated group exhibited an age-related reduction in body mass but a stable glucose level compared with the control group (Fig. 3f–g).

### Metformin inhibited particle-induced osteoclastogenesis in association with a reduction in the expression of osteoclastic genes

**Fig. 4:**
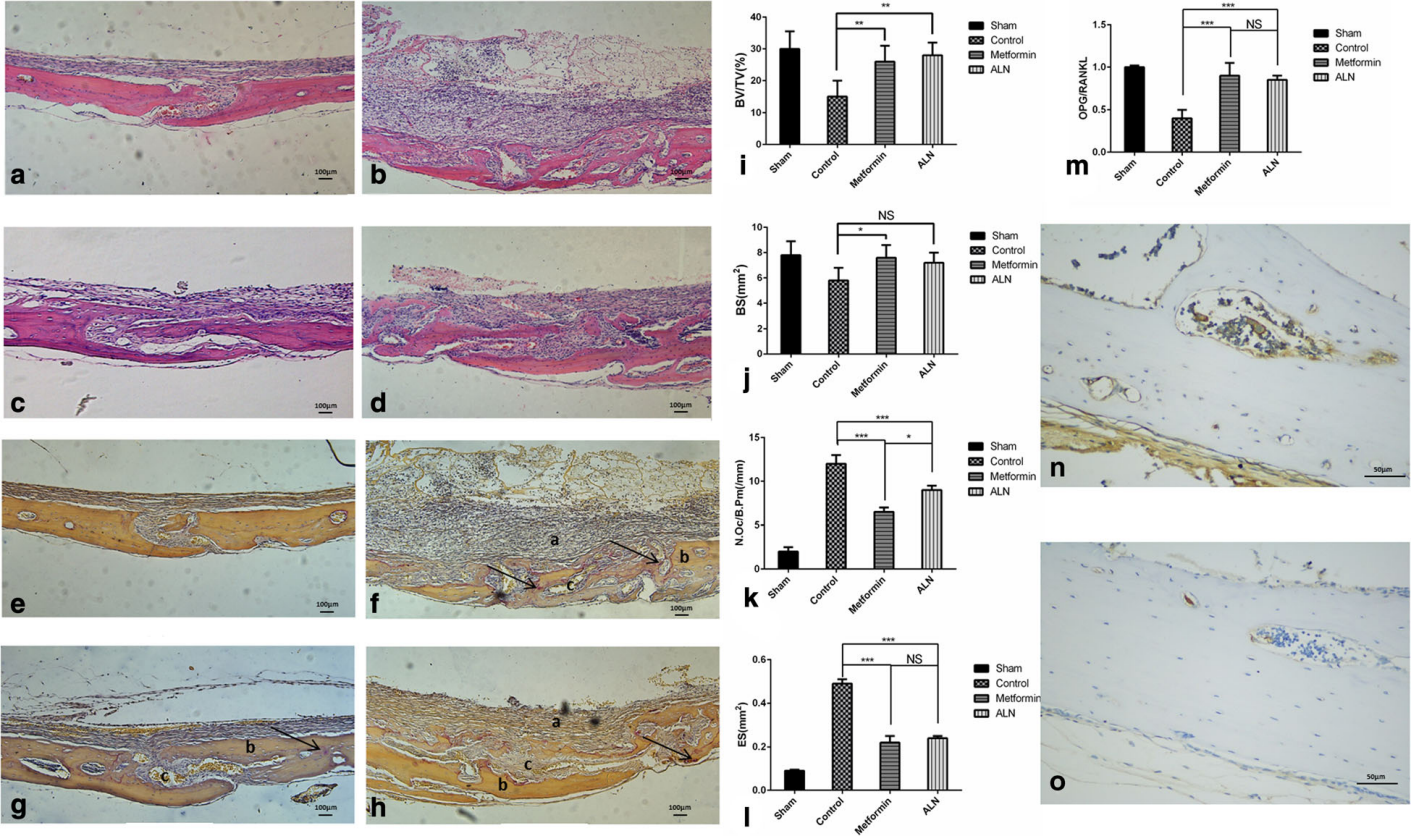
Representative images of histological sections of harvested calvaria for evaluation of inflammatory infiltration and associated osteolysis after implantation of UHMWPE particles. Compared to the sham control group (**a**), inflammatory infiltrate and osteolysis was observed in calvaria implanted with UHMWPE particles (**b**). The inflammatory response was suppressed by treatment with metformin (**c**) or ALN (**d**). Representative images of TRAP staining of mouse calvarial tissue from the different groups (**e**–**h**). a, inflammatory tissue, b, bone tissue, c, erosion area, arrow, osteoclasts. Quantitated bone histomorphometric analysis including BV/TV (**i**), bone surface area (**j**), average numbers of osteoclasts (**k**), and erosion surface area (**l**). **m** Expression of osteoclastic genes. Immunohistochemical staining for CD11b in control (**n**) and metformin-treated groups (**o**). Data are presented as means ± SD. ***P < 0.001; **P < 0.01; *P < 0.05; NS, *P* > 0.05

After particle implantation, histological staining of the harvested calvaria revealed an intense inflammatory infiltrate associated with osteolysis. Infiltrate resulting from an intense inflammatory response was revealed by histological staining of harvested calvaria with implanted particles. Fig. 4a shows the H&E stained tissue sections of the sham group. The osteolysis and fibrous tissue laden with the particles were observed in the H&E stained tissue sections (Fig. 4b). The histomorphometric data also suggested that the suture and osteolysis area increased from 0.082 ± 0.012 (sham group) to 0.406 ± 0.080 mm^2^ (particle-implanted group; *P* < 0.01) after particle implantation. The inflammation area decreased to 0.117 ± 0.010 mm^2^ with metformin treatment (*P* < 0.01) and 0.117 ± 0.010 mm^2^ with ALN treatment (*P* < 0.01; Fig. 4c, d). Via TRAP staining, osteoclasts were identified in the cytoplasm of multinuclear giant cells as purplish to dark red granules. TRAP staining confirmed the presence of osteoclasts in resorptive lacunae (Fig. 4e–h). Bone histomorphometric analysis showed reduced bone volume, bone surface area and increased erosion area in the control group. Metformin treatment reversed these changes (Fig. 4i–j, l). In the metformin- and ALN-treated groups, the mean numbers of osteoclasts per section (12.80 ± 3.03 and 18.60 ± 2.07, respectively) were significantly less than that in the particle-implantation group (22.7 ± 5.0, *P* < 0.01; Fig. 4k). One-way ANOVA revealed that the osteolysis area or the average number of osteoclasts were almost the same between the metformin- and ALN-treated groups. A considerable increase in the expression of osteoclastic genes in the metformin- and ALN-treated groups was observed (Fig. 4m). Immunohistochemical staining for CD11b showed more positive cells in the control group than in the metformin-treated group (Fig. 4n–o). Overall, the variation in osteolysis observed in the two-dimensional histological analysis was consistent with that observed by micro-CT, suggesting that metformin treatment decreased the osteolysis induced by the particles.

### Metformin inhibited UHMWPE particle-induced osteoclastogenenic cytokine production in vivo

**Fig. 5:**
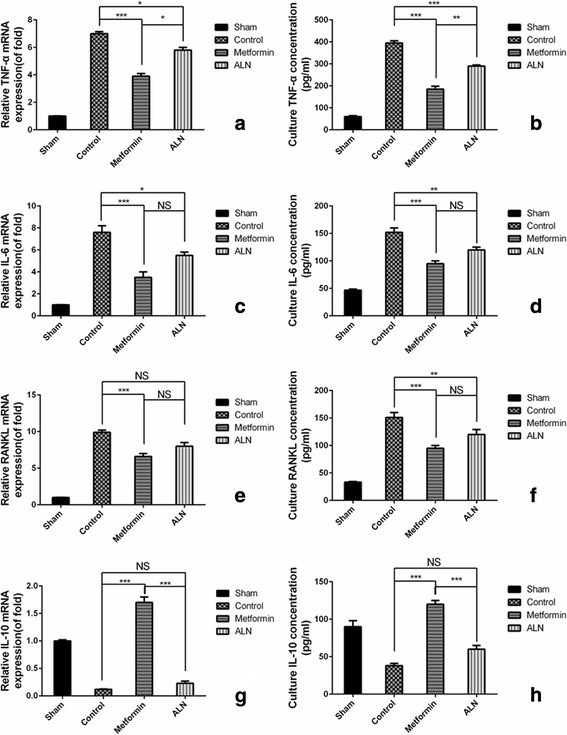
mRNA expression and secretion of cytokines by mouse calvarial tissue after implantation of UHMWPE particles and treatment with saline (control), metformin, or ALN. Relative (to that in the sham group) expression of TNF-α, IL-6, RANKL, and IL-10 (**a**, **c**, **e**, **g**, respectively) and secretion of TNF-α, IL-6, RANKL, and IL-10 (**b**, **d**, **f**, **h**, respectively) among the different treatment groups. ***P < 0.001; **P < 0.01; *P < 0.05; NS, *P* > 0.05

To study the mechanisms of the inhibitory effect of metformin on particle-induced osteolysis, the ability of metformin to suppress osteoclastogenic cytokine production induced by particle implantation was examined. The qRT-PCR results showed that particle implantation stimulated IL-6, TNF-α, and RANKL mRNA expression and suppressed IL-10 mRNA expression in calvaria. However, both metformin and ALN treatment inhibited these effects on cytokine production. Metformin presented potent effects on inflammatory cytokine release, whereas ALN strongly inhibited RANKL expression as compared to controls (*P* = 0.047; Fig. 5a, c, e, g). Analysis of the cytokine production in organ culture suggested that TNF-α, IL-6, and RANKL production were greatly increased upon exposure to particles. However, metformin and ALN treatment again significantly suppressed the particle’s effect on osteoclastogenic cytokine production. In addition, metformin treatment increased release of IL-10, whereas IL-10 secretion remained considerably lower in the ALN-treated group (*P* = 0.002 and *P* = 0.002, respectively; Fig. 5b, d, f, h). Consistent with the results of qRT-PCR, the results of calvaria culture also suggested that metformin had a potent effect on inflammatory cytokine production.

### Inhibitory effect of metformin on mouse calvarial osteolysis is dependent on promotion of macrophage polarization to M2 phenotype by AMPK activation

**Fig. 6:**
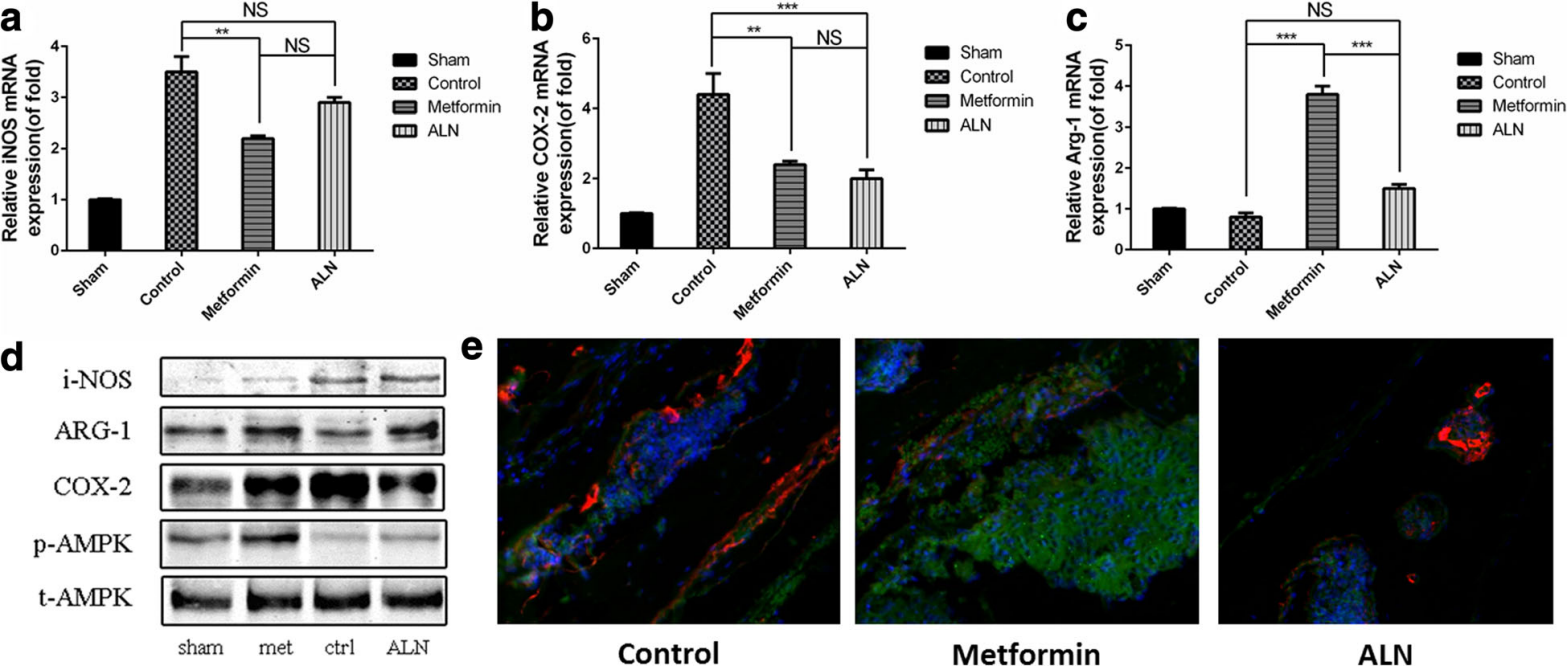
mRNA expression of proteins associated with macrophage phenotype and changes of protein expression according to western blot analysis in mouse calvarial tissue after implantation of UHMWPE particles and treatment with saline (control), metformin, or ALN. Relative mRNA expression of iNOS (**a**), COX-2 (**b**), and Arg-1 (**c**) among tissues from the different groups. **d** Protein expression of iNOS, COX-2, Arg-1, p-AMPK, and t-AMPK among tissues from the different treatment groups. **e** Double-labelling immunofluorescence of p-AMPK (green) and macrophage marker CD68 (red) in control group, metformin-treated group and ALN-treated group. (Cropped gels/blots are used here, and the full-length gels and blots are shown in Additional file 1: Figure S5.) ***P < 0.001; **P < 0.01; *P < 0.05; NS, *P* > 0.05

To determine whether metformin could promote macrophage polarization to an anti-inflammatory phenotype from a pro-inflammatory phenotype via AMPK activation, the effect of metformin on the expression of proteins related to macrophage functional phenotypes was investigated. The qRT-PCR results showed that particle implantation stimulated COX-2 and iNOs mRNA expression in calvaria (Fig. 6a–b). However, both metformin and ALN treatment inhibited these effects. Western blot analysis indicated that particle implantation caused increased expression of COX-2 from a relative expression ratio (relative to expression in the control group) of 0.594 to 1 and iNOS from 0.156 to 1, while metformin reduced the respective protein expression ratios from 1 and 0.799 to 1 and 0.308 (Fig. 6d). The increased COX-2 expression can be induced by inflammatory cytokines, and iNOS is expressed by M1 macrophages (Minghetti 2007). These results suggest that metformin inhibited the inflammation induced upon particle implantation in the mouse calvarial model. The qRT-PCR results and Western blot analysis also showed that metformin increased the expression of Arg-1, which is usually expressed by M2 macrophages (Fig. 6c–d). Moreover, the effect of metformin on the expression of proteins related to macrophage functional phenotypes depended on AMPK activation, as demonstrated by the increased expression of anti-inflammatory cytokines and the changes in the expression of phosphorylated AMPK. Double-labelling immunofluorescence of p-AMPK and CD68 showed more pAMPK and less CD68 positive cells in the metformin-treated group (Fig. 6e–f). These data imply that metformin inhibited mouse calvarial osteolysis by promoting macrophage polarization to the M2 phenotype via AMPK activation.

## Discussion

Osteolysis around orthopedic implants can develop via the formation of an inflamed periprosthetic membrane containing high levels of inflammatory cytokines, macrophages, and implant wear debris. Implant-derived wear particles can induce a unique immune response based on the particle size and concentration. The chemotactic cytokines secreted in response to UHMWPE particles can activate macrophages located in the tissues surrounding joint replacements (Frokjaer et al. 1995), creating an environment conducive to osteoclastogenesis and osteolysis. Bone marrow-derived macrophages may play dual roles in the osteolysis associated with TJA. One role is as the major cell type responsible for cytokine production in the host defense against UHMWPE particles, and the other is as the precursor cells for osteoclasts responsible for subsequent bone resorption. An additional study reported that the immune response to UHMWPE wear particles is characterized by cells of monocytic or osteoclastogenic lineage (Ren et al. 2011).

Macrophages produce varied arrays of mediators with pro- and anti-inflammatory capabilities in response to different tissue environments or stimuli (Malyshev and Malyshev 2015; Martinez et al. 2008). Macrophages expressing a pro-inflammatory versus an anti-inflammatory functional profile are distinguished using the designations M1 and M2 (Davies and Taylor 2015) . IL-10 is a regulating factor in macrophage polarization that inhibits the UHMWPE particle-induced increase in phospho-STAT1 and phospho-nuclear factor (NF)-κB p65 production and promotes phospho-STAT3 expression (Jiang et al. 2016). Therefore, manipulation of macrophage functional phenotypes can represent a potential therapeutic target for some diseases (Singh et al. 2014).

The AMPK pathway has emerged recently as a critical sensing mechanism in regulating cellular energy homeostasis and maintaining normal bone physiology (Patel et al. 2015; Wang et al. 2016). The bone mass in mice is decreased with deletion of the α or β subunit of AMPK (Shah et al. 2010; Quinn et al. 2010). Activation of AMPK stimulates bone formation in vitro (Kanazawa et al. 2008; Wang et al. 2016) and negatively regulates RANKL expression in the differentiation of osteoclasts (Kang et al. 2013). In addition, AMPK can promote macrophage polarization to an anti-inflammatory functional phenotype (Sag et al. 2008). Many studies have reported that AMPK phosphorylation was reduced in cells under the condition of inflammation or exposed to cytokines such as TNF-α (Vasamsetti et al. 2015). Activation of macrophages by UHMWPE particles induced inflammation and secretion of TNF-α, which reduced AMPK phosphorylation. Metformin has been discovered as a potent AMPK agonist that can suppress inflammatory cytokine production and osteoclast activation. Therefore, we hypothesized that it may be an effective drug against osteolysis induced by particles representing implant wear debris.

The effects of metformin on UHMWPE particle-induced production of inflammatory cytokines by primary mouse BMMs in vitro were examined. Incubation with the particles increased IL-6 and TNF-α production. Metformin not only inhibited the elevated production of IL-6 and TNF-α induced by UHMWPE particles, but promoted the release of the anti-inflammatory cytokine IL-10 and shifted polarization to M2 type in a dose-dependent manner. More importantly, the suppressed production of inflammatory cytokines was associated with phosphorylation of AMPK. Given the effects of metformin on both primary mouse BMMs and RAW264.7 cells as well as the fact that AMPK plays a significant role in the metabolism of both cell types, the results indicate that the effect of metformin on the production of macrophage-derived cytokines is dependent on AMPK activation.

Metformin attenuated the particle-induced mouse calvarial osteolysis, in association with a reduction in the severity of inflammation and osteoclast numbers. Consistent with in vitro results, activation of AMPK was involved in the protective effect of metformin against UHMWPE particle-induced osteolysis, via the promotion of macrophage polarization to an anti-inflammatory phenotype.

The anti-osteolysis effects of metformin and ALN as well-established leading drugs for treating osteoporosis (Black and Rosen 2016) show no significant difference, but their mechanisms are different. The classical pharmacological effects of ALNs are attributed to two key properties: the affinity for bone mineral and the ability to slow the activities of osteoclasts (Russell et al. 2008; Cantatore et al. 1999). Metformin, on the other hand, inhibited pro-inflammatory cytokine production, osteoclastogenesis, and osteolysis via the AMPK pathway. ALN cannot fundamentally inhibit osteolysis due to its limited capacity to inhibit the production of inflammatory cytokines and RANKL. For example, Zhang et al. (Zhang et al. 2005) reported that high local levels of TNF-α produced during periprosthetic inflammation prevented ALN-induced apoptosis among osteoclasts in vivo. Therefore, metformin should be more effective and useful than ALN in the treatment of periprosthetic osteolysis after TJA.

Oral administration of metformin hydrochloride (0.5–1.5 g) is routinely prescribed for the treatment of type II diabetes and has protective actions on the skeletal system (Monami et al. 2008; Hamann et al. 2012). According to our study, administration of 100 mg/kg metformin had a significant protective effect against the osteolysis induced by wear particles in the mouse calvarial osteolysis model. According to standard drug dose conversion between humans and animals, the dose of metformin used in this study is within the physiologically acceptable range. Since metformin can enhance insulin sensitivity and improve metabolic syndrome, it has been used not only for treating diabetes patients, but also for treating osteoporosis patients and individuals with metabolic syndromes barely affecting glucose levels. Many TJA patients are elderly and suffer from diabetes, which can cause osteoporosis (Voronov et al. 1998). Based on our results, we propose that long-term usage of metformin for treating type II diabetes might lessen the symptoms related to type II diabetes-related osteoporosis and offer some degree of protection against wear particle-derived periprosthetic osteolysis as well.

**Fig. 7:**
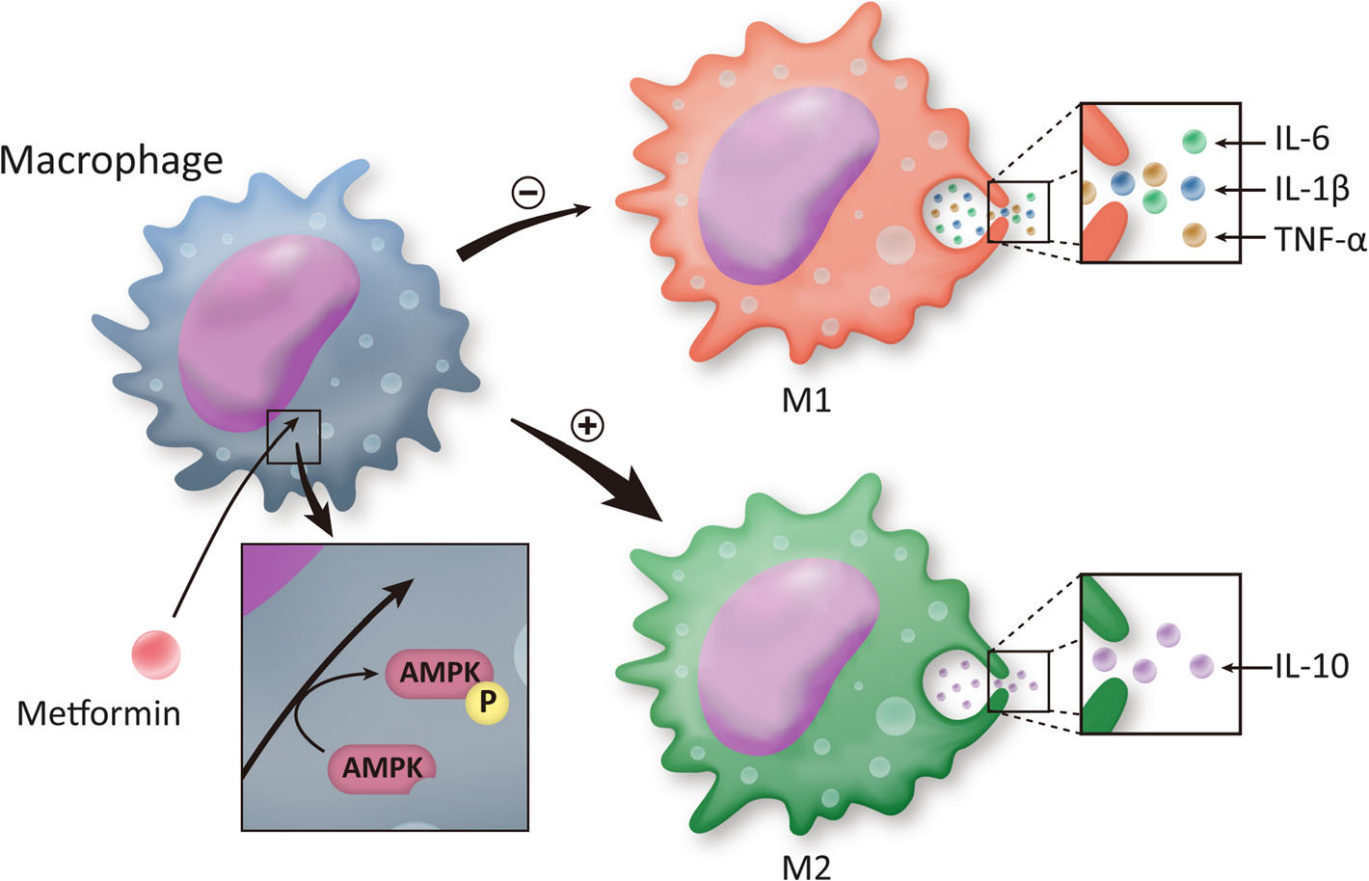
Metformin promoted polarization of macrophages to an anti-inflammatory phenotype via AMPK phosphorylation

In summary, as shown in Fig. 7, metformin remarkably inhibited the UHMWPE particle-induced polarization of mouse macrophages to the M1 phenotype, which is pro-inflammatory and characterized by iNos expression as a surface marker, and instead promoted the polarization of mouse macrophages to the M2 phenotype, which is anti-inflammatory and characterized by Arg-1 expression as a surface marker. As a result, the anti-diabetic drug inhibited the elevated production of IL-6 and TNF-α induced by UHMWPE particles and promoted the release of the anti-inflammatory cytokine IL-10 via the activation of AMPK in a dose-dependent manner. The results of the present study indicate that metformin inhibited the osteolysis in vivo induced by UHMWPE particles. These effects were found to be related to potent activation of AMPK.

The mechanism by which UHMWPE particles reduced the AMPK phosphorylation was not explored in our study, and the long-term effects of metformin administration on bone metabolism remain to be explored in our future experiments. Despite of the limitations of the present study, our work still indicates that metformin may represent a promising therapeutic agent for preventing or correcting periprosthetic osteolysis and implant loosening.

## Conclusion

Periprosthetic osteolysis and aseptic loosening are considered as proof of wear debris-induced disruption of local regulatory mechanisms related to excessive bone resorption associated with osteolysis and the damage at the bone-prosthesis interface. A crucial point in limiting and correcting the periprosthetic osteolysis and aseptic loosening is the inhibition of inflammatory factor production and osteoclast activation induced by activated macrophages. Metformin could limit the osteolysis induced by implant debris. The ability of metformin to attenuate osteolysis induced in mouse calvaria by the particles was related to a reduction in osteoclast number and polarization of macrophages to an anti-inflammatory functional phenotype.

## Additional file

Additional file 1: **Supporting Information S1.** Effects of Metformin on TNF-α, IL-6 and IL-10 production in RAW264.7. **Supporting Information S2.** Role of AMPK activation in the effect of metformin on the exposed in RAW264.7. **Supporting Information S3.** Characterizations. **Figure S1.** Effect of metformin on RAW264.7 cytokine production in response to UHMWPE particles and the effect on AMPK phosphorylation. **Figure S2.** Effect of metformin on RAW264.7 cytokine production with AICAR or Compound C in response to UHMWPE particles and the effect on AMPK phosphorylation. **Figure S3.** Full-length gels and blots of western blot results for phosphorylated AMPK (p-AMPK) and total AMPK (t-AMPK) in the different treatment groups. **Figure S4.** Full-length gels and blots of western blot results for phosphorylated AMPK (p-AMPK) and total AMPK (t-AMPK) in the different treatment groups with AICAR or Compound C. **Figure S5.** Full-length gels and blots of protein expression of iNOS, COX-2, Arg-1, p-AMPK, and t-AMPK among tissues from the different treatment groups. (DOC 1902 kb)
