# Supplementary material for: Metformin suppresses UHMWPE particle-induced osteolysis in the mouse calvaria by promoting polarization of macrophages to an anti-inflammatory phenotype
Source: Mol Med. 2018 May 9;24:20. doi: 10.1186/s10020-018-0013-x (PMC6016863; doi:10.1186/s10020-018-0013-x)
Supplement: Supplementary file 1 — Supporting Information S1. Effects of Metformin on TNF-α, IL-6 and IL-10 production in RAW264.7. Supporting Information S2. Role of AMPK activation in the effect of metformin on the exposed in RAW264.7. Supporting Information S3. Characterizations. Figure S1. Effect of metformin on RAW264.7 cytokine production in response to UHMWPE particles and the effect on AMPK phosphorylation. Figure S2. Effect of metformin on RAW264.7 cytokine production with AICAR or Compound C in response to UHMWPE particles and the effect on AMPK phosphorylation. Figure S3. Full-length gels and blots of western blot results for phosphorylated AMPK (p-AMPK) and total AMPK (t-AMPK) in the different treatment groups. Figure S4. Full-length gels and blots of western blot results for phosphorylated AMPK (p-AMPK) and total AMPK (t-AMPK) in the different treatment groups with AICAR or Compound C. Figure S5. Full-length gels and blots of protein expression of iNOS, COX-2, Arg-1, p-AMPK, and t-AMPK among tissues from the different treatment groups. (DOC 1902 kb) [file 10020_2018_13_MOESM1_ESM.doc]

**Additional file 1**

**Metformin Suppresses UHMWPE Particle-Induced Osteolysis in the Mouse Calvaria by Promoting Polarization of Macrophages to an Anti-Inflammatory Phenotype**

Zhao Yan^1, #^, Xiaoxi Tian^2, #^, Jinyu Zhu^1, #^, Zifan Lu^3^, Lifeng Yu^1^, Dawei Zhang^1^, Yanwu Liu^1^, Chongfei Yang^1^, Xiaorui Cao^1*^, Qingsheng Zhu^1*^

^1^ PLA Institute of Orthopaedics, Xijing Hospital, Fourth Military Medical University, Xi'an 710032, China; ^2^ Emergency department of Tangdu Hospital, Fourth Military Medical University, Xi'an 710038, China; ^3^ State Key Laboratory of Cancer Biology, Department of Pharmacogenomics, Fourth Military Medical University, Xi'an 710032, China.

^#^ Zhao Yan, Xiaoxi Tian, Jinyu Zhu contributed equally to this work

^*^ Corresponding authors: [caoxiaorui.810428@aliyun.com](mailto:caoxiaorui.810428@aliyun.com), [zhuqsh@fmmu.edu.cn](mailto:zhuqsh@fmmu.edu.cn)

**Content:**

Supporting Information S1: Effects of Metformin on TNF-α, IL-6 and IL-10 production in RAW264.7

Supporting Information S2: Role of AMPK activation in the effect of metformin on the exposed in RAW264.7

Supporting Information S3: Characterizations

Figure S1. Effect of metformin on RAW264.7 cytokine production in response to UHMWPE particles and the effect on AMPK phosphorylation.

Figure S2. Effect of metformin on RAW264.7 cytokine production with AICAR or Compound C in response to UHMWPE particles and the effect on AMPK phosphorylation.

Figure S3. Full-length gels and blots of western blot results for phosphorylated AMPK (p-AMPK) and total AMPK (t-AMPK) in the different treatment groups

Figure S4. Full-length gels and blots of western blot results for phosphorylated AMPK (p-AMPK) and total AMPK (t-AMPK) in the different treatment groups with AICAR or Compound C

Figure S5. Full-length gels and blots of protein expression of iNOS, COX-2, Arg-1, p-AMPK, and t-AMPK among tissues from the different treatment groups.

**Supporting Information S1: Effects of Metformin on TNF-α, IL-6 and IL-10 production in RAW264.7**

Production of IL-6, and TNF-α as pro-inflammatory mediators, as well as IL-10 as an anti-inflammatory mediator by RAW264.7 exposed to UHMWPE particles for 24 h was measured using ELISA kits. Within the incubation period of 24 h, the TNF-α and IL-6 levels in the media increased significantly (P<0.05), greater than those in the control cultures not exposed to particles (Fig. S1A-B). In contrast, the IL-10 levels in the culture media did not differ evidently between the groups (Fig. S1C). Treatment with metformin significantly reduced the increase in production of both TNF-α and IL-6, but enhanced the release of IL-10 after exposure to the particles for 24 h in a dose-dependent manner (Fig. S1A-C). ALN cannot inhibit the production of TNF-α and IL-6, neither can enhanced the release of IL-10.

**Supporting Information S2: Role of AMPK activation in the effect of metformin on the exposed in RAW264.7**

To investigate the dependence of metformin on cytokine production on AMPK activation, RAW264.7 was treated with AICAR to induce AMPK activation. In the same culture conditions, AICAR inhibited the particle-induced increases in TNF-α and IL-6 production and promoted IL-10 release in a concentration-dependent manner (Fig. S2A–C). Furthermore, as expected, AICAR treatment was associated with increased AMPK phosphorylation in a dose-dependent manner. AMPK was blocked next using compound C as a chemical inhibitor. The compound C not only limited the AMPK phosphorylation in a concentration-dependent manner, but clearly neutralized the effect of metformin on cytokine production (Fig. S2D-F). The changes in AMPK phosphorylation with AICAR treatment mirrored that the effect of metformin on cytokine release, thus, was highly related to AMPK activation in RAW264.7 with the particles.

**Supporting Information S3: Characterizations**

*Micro-CT analyses*

Before histological analysis, dissected samples fixed in paraformaldehyde were utilized for *in vitro* examination of the calvarial bone structure via high-resolution micro-CT (eXplore Locus SP; GE Healthcare, Madison, WI, USA). The voltage, current, and integration time were 80 kV, 80 mA, and 3000 ms, with a resolution of 14 mm, respectively. A region of interest (ROI) was selected as the largest osteolytic volume that fit in a 152×152×152 voxel template (85.2 mm^3^) [1,2]. The data analysis was performed using commercial software (MicroView ABA ver. 2.1.2, GE Healthcare). Three parameters, i.e., bone mineral content (BMC), bone mineral density (BMD), and the ratio of bone volume to tissue volume (BV/TV), were used to evaluate the particle-induced osteolysis.

*Histologic evaluation of osteolysis*

Harvested mouse calvaria samples were decalcified in 10% EDTA solution for histologic evaluation after micro-CT scanning was complete. An acid phosphatase kit from Sigma-Aldrich [3] and tartrate-resistant acid phosphatase (TRAP) stain were used to quantify osteoclasts, and the sagittal suture area was observed via hematoxylin and eosin (H&E) staining. The degree of osteolysis was evaluated in three neighboring sections under a microscope (Olympus BX 51, Tokyo, Japan) equipped with a video camera (Olympus DP72, Tokyo, Japan). A computer running the OsteoMeasure Analysis system (OsteoMetrics, Decatur, GA, USA) was attached. Each image was oriented with a midline suture of 1.3 mm in diameter in the field center and was captured at magnitude ×40. Soft tissues and the area of osteolysis were traced manually under 40× magniﬁcation in H&E stain slices, and quantiﬁed with the image analysis system mentioned above.

*Quantitative real-time polymerase chain reaction (qRT-PCR)*

Brain tissue was dissected from the bones, and the elliptical plate of bone, which is bound by the foramen magnum, auditory canals, and orbits, was removed carefully, to obtain the calvaria. According to the manufacturer’s suggestions, RNAs from calvaria specimens (including the soft tissue on the calvaria surface) were puriﬁed using Trizol reagent (Invitrogen, Carlsbad CA, USA). First-strand cDNA synthesis was catalyzed by murine leukemia virus (M-MLV) reverse transcriptase and performed using random primers (Invitrogen, Carlsbad, CA, USA). An ABI7500 system (Applied Biosystems, Foster City, CA, USA) was used to perform the real-time PCR. Each reaction volume contained the 5' primer (0.5μM), 3' primer (0.5 μM), SYBR Green I (10 μL, Takara, Dalian, China), sample (2 μL), and H_2_O to the final volume of 20 μL. For 45 cycles, the samples were amplified with a denaturation step at 95°C for 5 s, before the annealing and extension steps at 60°C for 34 s. The amount of double-stranded DNA was determined by measuring SYBR green fluorescence. A melting curve was generated after each run, to distinguish between the specific and nonspecific cDNA products. The relative mRNA levels of target genes were normalized to the β-actin levels and compared with those in control samples. The primers used were as follows: COX-2: F, 5’-GAAGTCTTTGGTCTGGTGCCTG-3’, R, 5’-GT CTGCTGGTTTGGAATAGTTGC-3’; iNOS: F, 5’-GGAGCGAGTTGTGGATTGTC-3’, R, 5’-GTGAGGGCTTGGCTGAGTGA-3’; IL-6: F, 5'-CTTGGGACTGATGCTGG-3', R, 5'-GGTCTGTTGGGAGTGGTAT-3’; TNF-α: F, 5'-TTCCCAAATGGCCTCCCT-3', R, 5'-TGGGCTACAGGCTTGTCACTC-3'; IL-10: F, 5'-GGGTTGCCAAGCCTTATCG-3', R, 5'-TCACTCTTCACCTGCTCCACT-3'; Arg-1: F, 5'-GGGAAGACAGCAGAGGAGGT-3', R, 5'-TAGTCAGTCCCTGGCTTATGG-3'; β-actin: F, 5'-TCCAGCCTTCCTTCTTGGGTAT-3', R, 5’-TGTTGGCATAGAGGTCTTTACGG-3’.

*Calvaria culture*

The calvaria (including the soft tissue on the surface) of five mice were removed en bloc under sterile conditions and assigned for culture randomly. Each calvaria was transferred to a well of a 12-well plate, and then serum- and phenol-free Dulbecco’s modified Eagle’s medium (DMEM; 1 mL), supplemented with 1% penicillin, streptomycin, and glutamine (Invitrogen, Paisley, UK) was added for culture at 37°C in 5% CO_2_. After 24 h, the culture medium was collected and stored at -80°C for later measurement of the TNF-α, IL-6, IL-10, and RANKL concentrations. Subsequently, calvaria were calcinated, and the ashes were weighed for normalization of the production of cytokines.

*Immunohistochemical staining*

Before staining, paraffin embedded tissue microarray sections were deparaffinized and then hydrated through graded concentrations of alcohol. For antigen retrieval, sections were incubated with citrate buffer (pH 6.0, 10 mM) in a pressure cooker for 30 min. The endogenous peroxidase activity was cleared using 3 % H_2_O_2_ in methanol for 10 min. The sections were incubated in BSA (Servicebio, China) for 30 min. Anti-CD11b antibody MAB1124-SP (R&D Systems, USA) was used at a dilution of 1:500. Normal rabbit IgG was used at the same dilution as the negative control. After incubation with the primary antibody or rabbit IgG overnight at 4 °C, the sections were washed in PBS and incubated with biotinylated goat anti-rabbit IgG at a dilution of 1:200 and VECTASTAIN® ABC Reagent (Vectastain Elite ABC kit; Vector Laboratories) at a dilution of 1:400 consecutively for 30 min each. Peroxidase activity was demonstrated by adding DAB as a chromogen (Sigma). The sections were then counterstained with hematoxylin, dehydrated through alcohol gradient and examined under the light microscope.

*Flow Cytometry*

Cells were fixed with 2 % paraformaldehyde and permeable reagent (eBioscience) for 30 min on ice. Cells were stained with fluorescently labeled antibodies (1 μg/ml; 1 h at room temperature), assayed by flow cytometry (FASCAria II, BD Bioscience, Franklin Lakes, NJ, USA), and data were analyzed by Flowjo7.6 software (Tree Star, Inc. Ashland, OR, USA). Primary antibodies used for staining were anti-mouse cd206 (eBioscience),

*Western blot analysis*

Expression of inducible arginase 1 (ARG-1), nitric oxide synthase (iNOS), phosphorylated AMPK, and cyclooxygenase 2 (COX-2) was measured in calvaria specimens (including the soft tissue on the surface). First, calvaria specimens were lysed in radioimmunoprecipitation (RIPA) buffer, i.e.,Tris–HCl pH 7.4 (0.05 M), NaCl (0.15 M), Nonidet P-40 (1%), deoxycholic acid (0.25%), , EDTA (1 mM), aprotinin (1 mg/ml), phenylmethylsulfonyl ﬂuoride (1 mM), and leupeptin (1 mg/ml). The BCA protein assay was utilized to measure the total protein concentration. Before transfer to a nitrocellulose membrane, sodium dodecyl sulfate (SDS)-polyacrylamide gel electrophoresis (PAGE) was used to separate the proteins. The membrane was blocked by a 10% solution of skim milk at room temperature for 2 h and incubated at 4°C for overnight with the prepared primary antibody solutions, including anti-ARG-1, anti-iNOS, anti-COX-2, or anti-phospho-AMPK(Thr172) (Cell Signaling Technology Inc., Beverly, MA). After three washes with Tris-buffered saline containing Tween-20 (TBST), the membranes were incubated for 1 h with the appropriate secondary antibody [goat anti-mouse or goat anti-rabbit IgG (0.1 μg/ml) coupled to infrared (IR) dyes] in TBST at room temperature without light. After rinsing, the Odyssey IR imaging system (Li-COR Biosciences, Lincoln, NE, USA) was used to visualize the resulting bands.


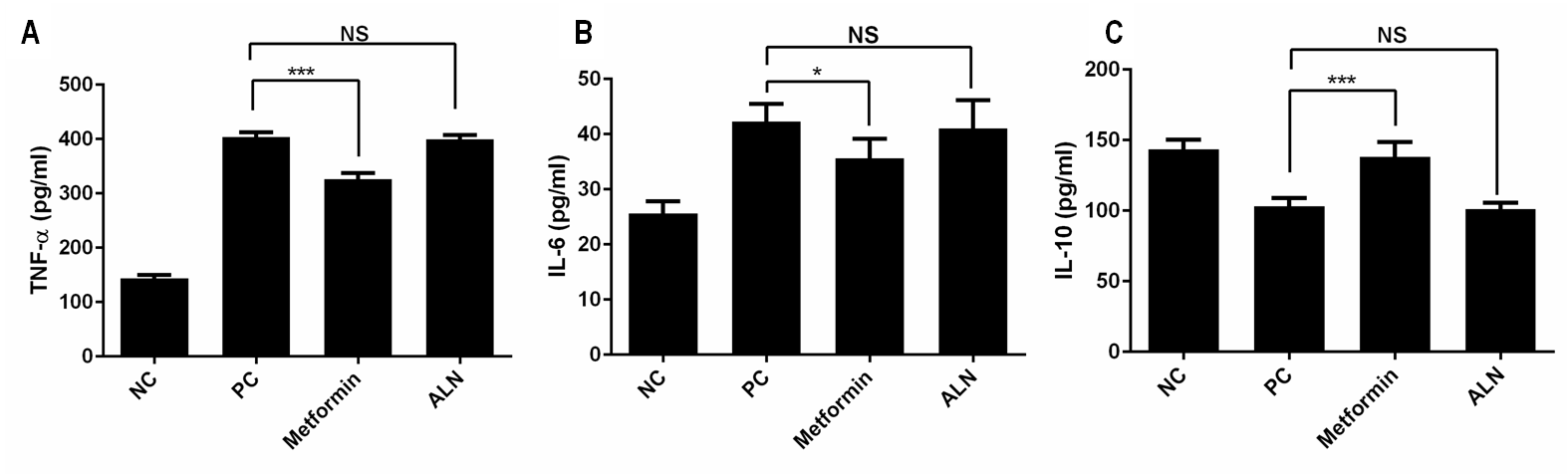


Figure S1. Effect of metformin on RAW264.7 cytokine production in response to UHMWPE particles and the effect on AMPK phosphorylation. Concentrations of (A) TNF-α, (B) IL-6, and (C) IL-10 in culture media after exposure of the cells to UHMWPE particles and 5 mM of metformin as well as 0.01μM ALN. Data represent the means ± SD. NC, negative control (no treatment); PC, positive control (treatment with UHMWPE particles only). ***P < 0.001; **P < 0.01; *P < 0.05.


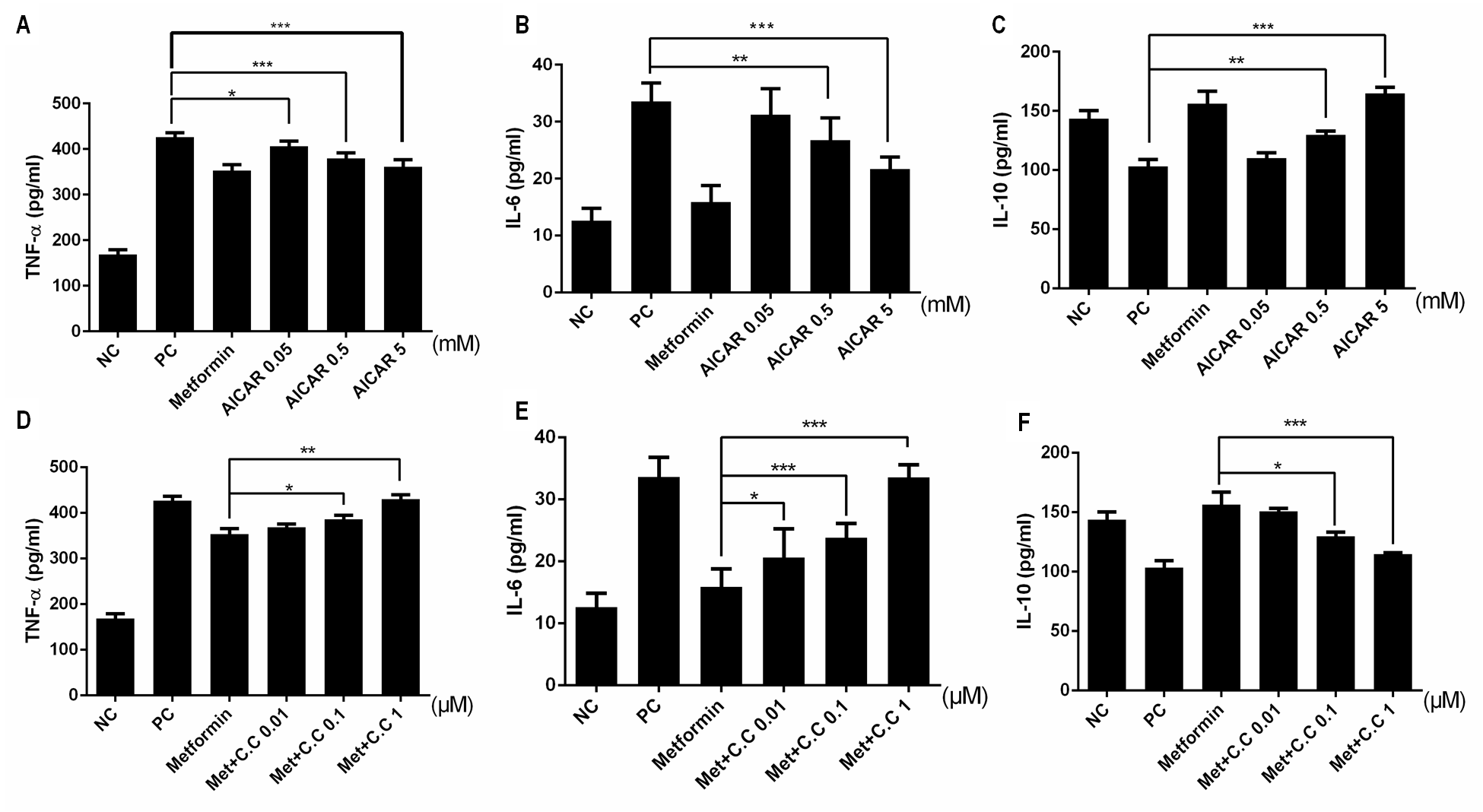


Figure S2. Effect of metformin on RAW264.7 cytokine production with AICAR or Compound C in response to UHMWPE particles and the effect on AMPK phosphorylation. Concentrations of (A) TNF-α, (B) IL-6, and (C) IL-10 in culture media after exposure to UHMWPE particles, metformin (5 mM), and different concentrations of AICAR (0.05, 0.5, or 5 mM) to activate AMPK. Concentrations of (D) TNF-α, (E) IL-6, and (F) IL-10 in culture media after exposure to UHMWPE particles, metformin (5 mM), and different concentrations of Compound C (0.01, 0.1, or 1 µM) to block AMPK. Data represent the means ± SD. NC, negative control (no treatment); PC, positive control (treatment with UHMWPE particles only). ***P < 0.001; **P < 0.01; *P < 0.05.


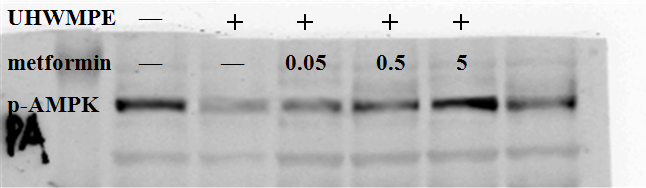


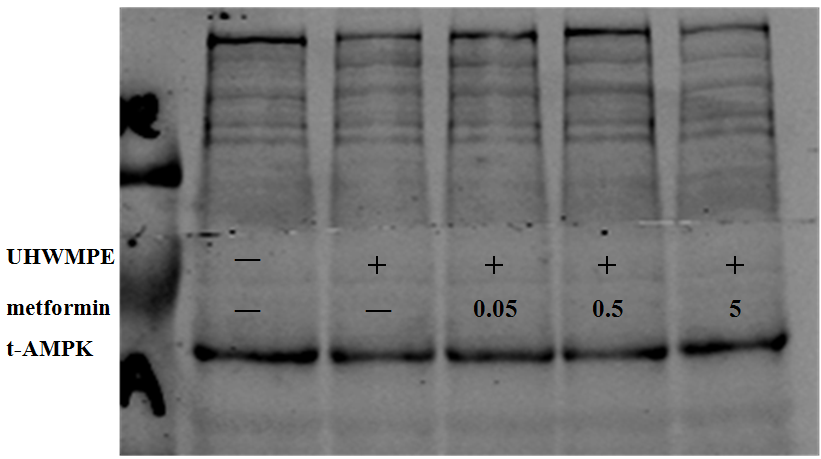


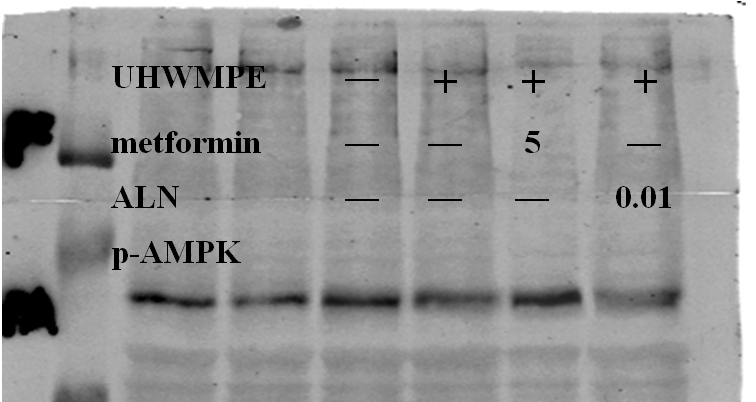


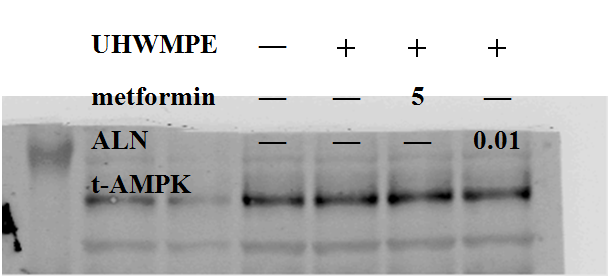


Figure S3. Full-length gels and western blots for phosphorylated AMPK (p-AMPK) and total AMPK (t-AMPK) in the different treatment groups.


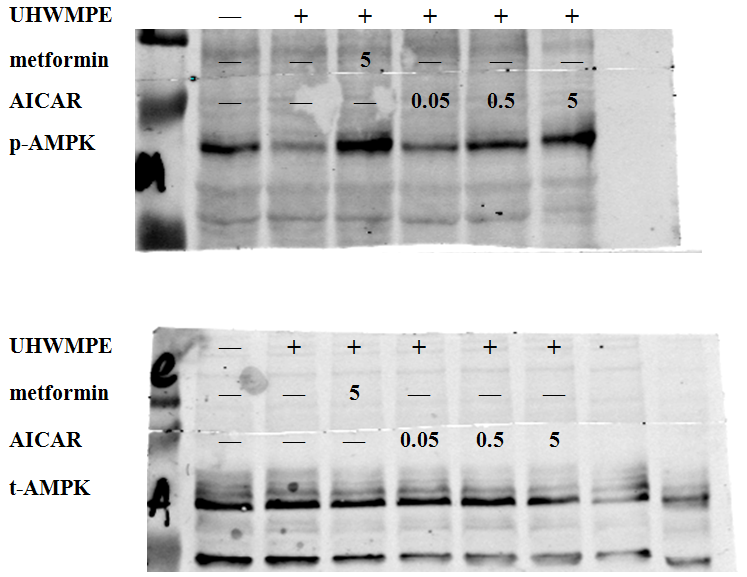


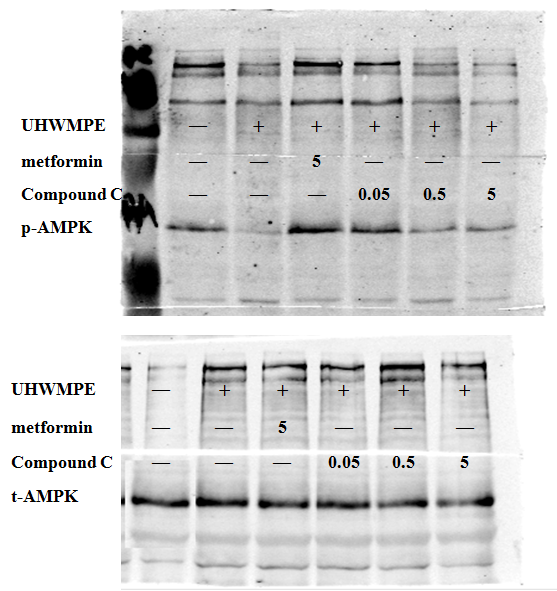


Figure S4. Full-length gels and western blot for phosphorylated AMPK (p-AMPK) and total AMPK (t-AMPK) in the different groups treated with AICAR or Compound C.


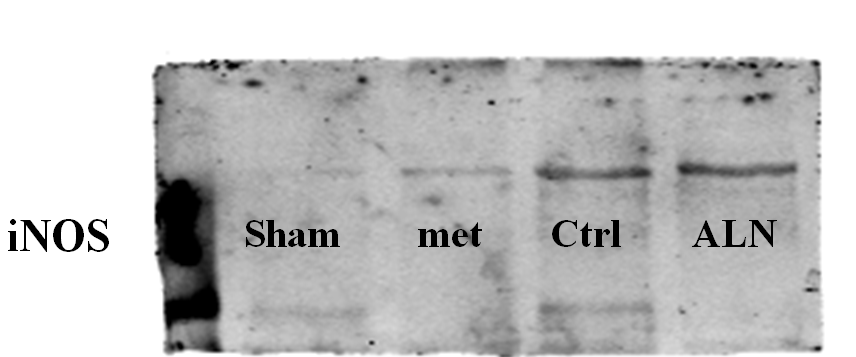


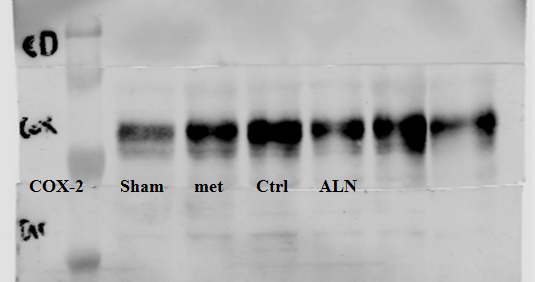


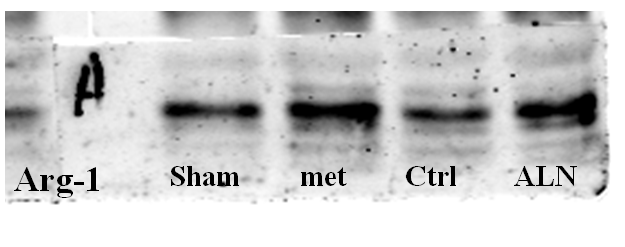


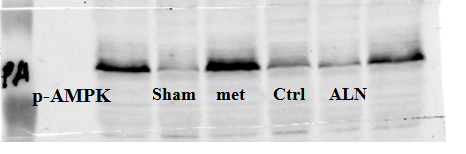


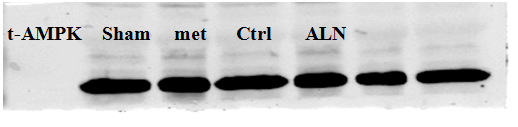


Figure S5. Full-length gels and blots showing protein expression of iNOS, COX-2, Arg-1, p-AMPK, and t-AMPK among tissues from the different treatment groups.

**References:**

1. Tsutsumi, R. *et al.* Differential effects of biologic versus bisphosphonate inhibition of wear debris-induced osteolysis assessed by longitudinal micro-CT. *J ORTHOP RES* **26**, 1340-1346 (2008).

2. Yang, C. *et al.* The aqueous extract of Angelica sinensis, a popular Chinese herb, inhibits wear debris-induced inflammatory osteolysis in mice. *J SURG RES* **176**, 476-483 (2012).

3. Schwarz, E.M. *et al.* Quantitative small-animal surrogate to evaluate drug efficacy in preventing wear debris-induced osteolysis. *J ORTHOP RES* **18**, 849-855 (2000).
